# Too Wise to Learn from Books

**Published:** 1842-03

**Authors:** 


*Too wise to learn from books.*—An itinerant dentist of a neighboring state was asked a short time since, if he was a subscriber to the "American Journal of Dental Science." He said "no, I doesen't read books, I depends on occular demonstration." We very much question whether he could read, if he had the inclination. But, notwithstanding the ignorance of this individual, we are told that he has sense enough to impose upon many intelligent people, and we need not wonder at the degraded state of the profession when such men are patronized. If people will suffer themselves to be thus easily duped, they are not much to be pitied.

